# Characterization of C-ring component assembly in flagellar motors from amino acid coevolution

**DOI:** 10.1098/rsos.171854

**Published:** 2018-05-09

**Authors:** Ricardo Nascimento dos Santos, Shahid Khan, Faruck Morcos

**Affiliations:** 1 Institute of Chemistry and Center for Computational Engineering and Science, University of Campinas, Campinas, SP, Brazil; 2 Molecular Biology Consortium, Lawrence Berkeley National Laboratory, Berkeley, CA, USA; 3 Department of Biological Sciences, University of Texas at Dallas, Richardson, TX, USA; 4 Department of Bioengineering, University of Texas at Dallas, Richardson, TX, USA; 5 Center for Systems Biology, University of Texas at Dallas, Richardson, TX, USA

**Keywords:** flagellar motor, bacterial motility, coevolution, structure-based model

## Abstract

Bacterial flagellar motility, an important virulence factor, is energized by a rotary motor localized within the flagellar basal body. The rotor module consists of a large framework (the C-ring), composed of the FliG, FliM and FliN proteins. FliN and FliM contacts the FliG torque ring to control the direction of flagellar rotation. We report that structure-based models constrained only by residue coevolution can recover the binding interface of atomic X-ray dimer complexes with remarkable accuracy (approx. 1 Å RMSD). We propose a model for FliM–FliN heterodimerization, which agrees accurately with homologous interfaces as well as *in situ* cross-linking experiments, and hence supports a proposed architecture for the lower portion of the C-ring. Furthermore, this approach allowed the identification of two discrete and interchangeable homodimerization interfaces between FliM middle domains that agree with experimental measurements and might be associated with C-ring directional switching dynamics triggered upon binding of CheY signal protein. Our findings provide structural details of complex formation at the C-ring that have been difficult to obtain with previous methodologies and clarify the architectural principle that underpins the ultra-sensitive allostery exhibited by this ring assembly that controls the clockwise or counterclockwise rotation of flagella.

## 1. Introduction

**Figure 1.**
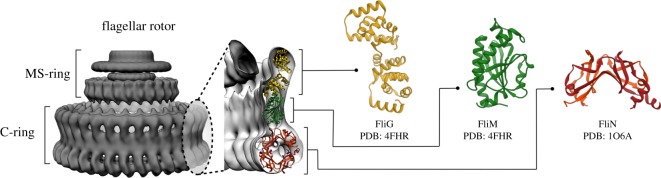
Architecture of flagellar rotor [4]. Proteins FliG, FliM and FliN are the primary building blocks of the C-ring in the basal body of flagella.

Flagellar motors (FM) are intricate molecular machines fundamental for bacterial motility. These motors, embedded in the basal body, are powered by electrochemical ion gradients. Transmembrane stator complexes couple ion flux to bidirectional rotation. Modulation of the clockwise (CW)/counter-clockwise (CCW) rotation bias by chemotactic stimuli drives migration in chemical gradients through a biased random walk [1]. CCW and CW intervals as measured in cells tethered by a single flagellum have mean durations of a second or so, but the cells switch between rotation states within milliseconds without detectable changes in rotation speed. Torque is generated when the complexes, acting independently, step along a FliG protein rotor ring attached to the transmembrane MS-ring scaffold [2,3]. A large multi-subunit cytoplasmic ring assembly, the C-ring (figure 1), also forms part of the rotor [3,5,6]. In the eubacteria, *Escherichia coli* and *Salmonella typhimurium*, the C-ring is composed of the FliM and FliN proteins (figure 1). The C-rings of other bacteria contain FliY instead of, or in addition to, FliN [7]. The chemotaxis signal protein CheY binds and tethers to the FliM N-terminal domain to trigger long-range allosteric changes that result in a large reorientation of the FliG helix contacted by the stator complexes and, thereby, rotation reversal [8]. The change in rotation bias is an ultra-sensitive function (Hill coefficient of 10.5) of activated CheY concentration [9,10]. There are about 35 copies of FliM and about three times that number for FliN [11]. The C-ring is a dynamic structure. It loses or gains FliM and FliN subunits to adaptively reset rotation bias upon chemotactic stimulation [12,13]. Accordingly, the subunit symmetry of the C-ring is also variable, but the mean number is 34 [4,14]. Conformational spread across subunits must occur to switch C-ring state within milliseconds [15]. These characteristics emphasize that switching of flagellar rotation provides a remarkable example of long-range allostery whose elucidation will be an important advance for protein science.

A library of X-ray atomic structures for the FliG, FliM and FliN proteins is available, and ring models of their organization have been constructed based on fits to electron density maps (figure 1) [4,3]. The models have been guided by mutagenesis, *in situ* cross-linking and mass spectrometry data [16–18]. More recently, computational methods, particularly residue coevolution, have guided model-building of flagellar protein complexes generally [19] and in FliG specifically [20]. The coevolution signal for FliM is distinct from the FliG middle domain. Inter-subunit contacts make a strong contribution to the signal for the latter, consistent with its proposed central location in the C-ring and its role in conformational spread [21]. There are competing models for the distal C-ring. One model proposes a periodic repeat of a 4:1 complex of FliN C-terminal homo-tetramer with a FliM C-terminal domain (FliN_*C*_ and FliM_*C*_) [22]. Another model proposes a repeat of a 3:1 complex with a FliN_*C*_ homodimer juxtaposed with a FliM_*C*_–FliN_*C*_ heterodimer [16]. Both models are consistent with the sequence homology between FliM_*C*_ and FliN_*C*_ as well as *in situ* cross-link data. The coevolutionary signal for the distal C-ring has not been determined thus far.

Here, we present models for the architecture of FliM middle domain (FliM_*M*_) and FliM_*C*_–FliN_*C*_ complexes that address the above issues. Our study is based on the analysis of amino acid coevolution [23–30]. Recent applications of coevolution have focused on its use as a probe of protein structure, conformational states and dynamics [25,31–48]. To study coevolutionary signals, we use direct coupling analysis (DCA), an approach advanced by us and others, for a number of years [24,25,49–52]. Most recently, we used residue coevolution, including DCA together with dynamics extracted from conformational ensembles based on bonding constraints, to detect alternative conformational states of FliM_*M*_ [53]. The ensemble approach retains full-atom detail, but lacks temporal information [54]. In this study, we apply structure-based models (SBM) to rigorously evaluate the FliM_*M*_ conformational heterogeneity detected by coevolutionary signals. Structure-based models provide a particularly elegant approach based minimally on heavy atom volume exclusion to evaluate coevolutionary constraints [55–57]. We demonstrated in a previous study that SBMs were effective in predicting complex formation from monomer X-ray structures when coevolved couplings are employed as distance constraints to drive MD simulations [45]. Most of these studies have been focused on validating the approach rather than providing new biological insights into the systems studied. This work aims to be an example of how coevolutionary signals and physical modelling can bring a more detailed understanding of the molecular interactions shaped by evolution and at the same time be consistent with low-resolution experimental evidence on the C-ring complexes. We report that the coevolutionary signal for distal C-ring components is enough to reproduce FliN_*C*_ homo-dimer association within 1 Å root mean square deviation (RMSD) and to predict FliM_*C*_–FliN_*C*_ heterodimers. Moreover, two distinct dimer conformations for FliM_*M*_ were obtained in the absence of chemical bonding constraints from just coevolutionary constraints and the monomer X-ray structure. These dimer states precisely agree with *in situ* cross-linking data and are inter-convertible in a way that is consistent with switching kinetics for flagellar rotation.

## 2. Material and methods

### 2.1. Protein sequence datasets

For FliN_*C*_ and FliM_*C*_ proteins, 11 119 sequences were obtained from the Uniprot database and aligned using hidden Markov models (HMM) provided in the Pfam server for FliMN_*C* domain (family PF01052) [58,59]. The extended version of FliN domain multiple sequence alignment (MSA) was generated using the full sequence of *Thermotoga maritima* FliN (Uniprot G4FDE4) with the HMMER server, selecting only those sequences that have a protein architecture with both FliM and FliMN_C domains [60]. This procedure generated a MSA with 5151 sequences. With respect to FliM, we built a custom MSA using the HMMER server [61]. A HMM profile was created using the FliM sequence of *T. maritima* (Uniprot Q9WZE6) and selecting only sequences that are components of the bacterial flagellum. Sequences with more than 30 consecutive mispairing positions (gaps) were removed, accounting for approximately 15% of the dataset. The resulting MSA used for DCA is composed of 3939 sequences.

### 2.2. Coevolutionary analysis, sequence mapping and contact selection

Each MSA was processed using the mean field version of direct coupling analysis (mfDCA) [24,25,34]. We model the joint probability of a given protein sequence to be part of a family with a statistical model resulting from entropy maximization.

In order to infer the parameters of these distributions, we use an efficient mean field approach [25]. This approach estimates the parameters of a global probability distribution of amino acid occupancies. The parameters in the distribution can be used to measure how two sites are directly coupled in the family and to infer potential residue–residue interactions. A pairwise ‘direct’ probability is computed and used in a metric called direct information (DI):

2.1 $${\mathrm{DI}}_{ij}=\sum_{A,B}P_{ij}^{\left(\mathrm{dir}\right)}(A,B)\ln\frac{P_{ij}^{\left(\mathrm{dir}\right)}(A,B)}{f_{i}(A)f_{j}(B)},$$

where *A* and *B* denote two amino acid types appearing at positions *i* and *j* of the MSA (i.e. the *i* and *j* columns in an alignment matrix) [25]. In addition, *f*_*i*_(*A*) denotes the frequency at which *A* is observed at position *i* of the alignment and $P_{ij}^{(\mathrm{dir})}$ computes the pairwise probability of direct correlation [25]. Highly coupled residue pairs ranked by DI values represent pairwise amino acids that plausibly coevolved to satisfy physical requirements to structure and function (i.e. folding, oligomerization, conformational dynamics). The final residue–residue interaction map was generated by domain matching in protein sequences with HMMER, employing an in-house developed mapping script. In order to identify signals that will exclusively drive our predictions towards oligomerization, we discard interdomain folding contacts by combining solvent accessibility and crystallographic data [45]. This step comprises the use of all-atom monomeric X-ray structures from *Thermotoga maritima* (PDB IDs: 4FHR chain A for FliM_*M*_ and 1O6A chain A for FliM_*C*_ and FliN_*C*_) to compute the solvent accessible surface area (SASA) of individual residues employing GetArea [62]. In each case, from that list of top coevolving DCA pairs, only those pairs for which the summation of SASA values was higher that 50% were considered for modelling studies. For the FliM_*C*_−*N*_*C*_ dimer prediction, due to the lack of a crystallographic data for FliM_*C*_ and the fact this system is a heterodimer, we filtered contacts by finding equivalent DCA contacts for FliN_*C*_ generated by monomeric filtering and remapping to FliM_*C*_ model. The selection of DCA contacts for prediction of oligomeric complexes involving FliN_*C*_ and FliM_*C*_ (tetramers in the complete ring) was performed by employing a scheme that removes all contacts between DCA pairs that were already in contacts at the predicted dimers. Contact maps were computed considering C*α* atom pairs within a distance of 10 Å. In order to compute the interfacial RMSD for the predicted *T. maritima* FliM_*C*_–FliN_*C*_ heterodimer, the *T. maritima* sequences were aligned to the reference chimeric *Salmonella enterica* sequence (PDB ID: 4YXC), and matching inter-protein pairs within 10 Å were considered in the calculation.

### 2.3. Structure-based model simulations

The coevolutionary constraints obtained after mapping and filtering DCA pairs were incorporated as Gaussian-like potentials to drive complex formation along SBM MD simulations [63]. An iterative process was used that progressively reduces the equilibrium distance of the constraints to allow proper exploration of the dimeric interface space [45]. Initially, PDB coordinates with separate copies of the monomeric structures for each system were used as input to the SMOG server [56]. The parameter and topology files generated by SMOG were then modified by inserting the SBM potentials generated from the predicted coevolutionary constraints. Finally, MD simulations were developed using a modified version of GROMACS software including support to SBM Gaussian-like potentials [64,63]. The final interaction potential for each residue pair is given below.

2.2 $$V_{ij}(r_{ij})=A_{ij}\left[\left(1+\left(\frac{1}{A_{ij}}\right)R_{ij}(r_{ij})\right)(1+G_{ij}(r_{ij}))-1\right],$$

where *R*_*ij*_(*r*_*ij*_) accounts for volume exclusion repulsion factor and is given by

2.3 $$R_{ij}(r_{ij})=\epsilon {\left(\frac{d}{r_{ij}}\right)}^{12},$$

where *d* and *ϵ* are the exclusion volume and a normalization constant, respectively. *G*_*ij*_(*r*_*ij*_) is the Gaussian attractive interaction:

2.4 $$G_{ij}(r_{ij})=-\epsilon \exp\left[-\frac{{\left(r_{ij}-r_{ij}^{o}\right)}^{2}}{\left(2w_{ij}^{2}\right)}\right].$$

These Gaussian-like potentials allow us to define parameters such as the interaction force amplitude (*A*_*ij*_) and width (*w*_*ij*_) independently of the optimal distance (*r*^*o*^_*ij*_) defined for each pair *i* and *j* [63].

The final C*α* coordinates of each model were used to reconstruct all-atom models by including side-chain optimized rotamers with REMO [65]. For FliM_*C*_–FliN_*C*_ heterodimer, the monomeric model for FliM_*C*_ was generated through the SwissModel server [66] using the FliM_*C*_ sequence for *T. maritima* and a crystallographic FliN_*C*_ model as a template (PDB ID: 1O6A, chain A).

Because the complexes in this work were obtained by using one domain family, the predicted pairs present a unique configuration in which residues *i* and *j* are represented by the first and second indexed monomeric chain of the system, respectively. In order to consider the possibility of residue *j* in the first chain interacting with *i* in the second, we also included a second specular constraint map (*j* and *i*) to generate the SBM potentials. To perform the step-by-step approximation between monomers during simulations, we started the system with two distant and randomly oriented separated monomers and progressively changed the parameters of equilibrium distance (*r*) and potential width (*w*) from SBM potentials. This procedure was performed in MD simulation steps of 10 ns using the following combination of parameters (*r*/*w*): 50 Å/2, 30 Å/2, 15 Å/4, 11 Å/4, 6.4 Å/4, 6.4 Å/2 and 6.4 Å/0.5. All steps were performed at a relative temperature of 120 (normalized in SMOG implementation, see [56]). The reduction in the Gaussian width constant (*w*) in the last step helps to refine the interface and to optimize the accuracy of the complex modelling. Furthermore, in order to isolate the real contribution of DCA pairs in complex predictions, we performed MD simulations for negative controls. These control simulations are further detailed in the electronic supplementary material.

## 3. Results

### 3.1. Coevolutionary signals recapitulate FliN dimerization and help model lower C-ring formation

**Figure 2.**
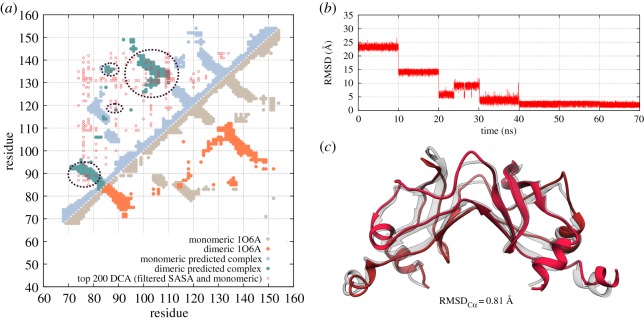
Prediction of the FliN_*C*_ homodimer. (*a*) Comparison between residue contacts in the crystal structure (above the diagonal in blue circles) and the DCA-predicted structure (lower diagonal in orange circles) for FliN dimerization. Interactions predicted from DCA and used to generate the FliN complex from FliN monomers are also shown (red circles, upper left corner). (*b*) RMSD between the predicted FliN_*C*_ homodimer and the crystal structure used as reference (PDB ID: 1O6A) for the simulation. (*c*) The best predicted model (in red) presented a RMSD of 0.81 Å when compared to the reference model (in white).

As a way to explore the feasibility of using coevolutionary signals to investigate complex formation in families of Fli proteins, we analysed sequences of proteins from FliM and FliN families using DCA [25,50] to predict C-ring molecular complexes [45]. In an initial step, employing exclusively unbiased coevolutionary signals and physically separated monomeric structures, we predicted the association of a FliN_*C*_ homodimer from *T. maritima* and compared it with reported structures for this complex (PDB ID: 1O6A). We analysed sequences in the family for the C-terminal domain shared by both FliM and FliN proteins (FliMN_*C*, Pfam PF01052) using DCA (see Material and methods section). This analysis allowed us to observe high coevolutionary signals for FliN by the identification of populated contact regions superposing to monomeric contacts and captured in the top-ranked DCA correlations (electronic supplementary material, figure S3). Figure 2*a* shows the top DI couplings for a MSA of this dataset after the application of a filtering process to remove contacts related to folding and to isolate contacts mainly originating from dimerization (see Material and methods section). When compared to the native contacts from a crystal structure of a FliN_*C*_ homodimer, it is possible to observe representative clusters of coevolving residue pairs that coincide with dimeric regions (dashed circles in figure 2*a* upper-left corner). These residue pairs (200 pairs with highest coevolving signal and filtered for solvent accessibility) were used to build a physical potential in coarse-grained molecular dynamics (MD) simulations for the prediction of the homodimeric FliN complex (figure 2*b*). In this process, two copies of the X-ray crystallographic structure of chain A from FliN (PDB ID: 1O6A) were used to predict a homodimer. This methodology takes advantage of the cooperative nature of native interactions and is robust to noisy predicted interactions (see controls in electronic supplementary material, figures S1 and S2). Using this procedure, we were able to recapitulate with high accuracy the correct FliN homodimer conformation, with a lowest RMSD of only 0.81 Å (figure 2*c*; electronic supplementary material, Movie S1) and an average RMSD of 1.97 Å over the last simulation stage (see Material and methods section). Representative DCA pairs used as potentials to drive dimer association are shown in the three-dimensional depiction in electronic supplementary material, figure S4.

### 3.2. Characterization of the FliM–FliN interaction interface

**Figure 3.**
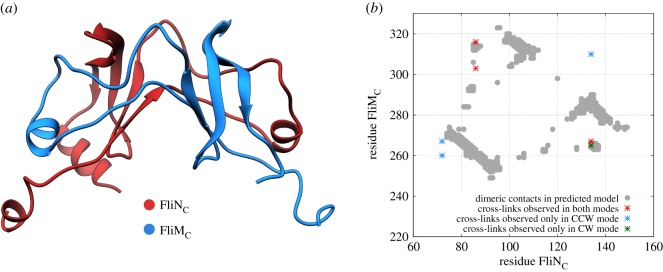
Prediction of the FliM_*C*_–FliN_*C*_ heterodimer. (*a*) Three-dimensional structure of the predicted heterodimer. (*b*) Comparison of dimeric contacts for the predicted FliM_*C*_–FliN_*C*_ heterodimer that were observed as cross-links [22]. Good agreement between the model contacts and cross-links is observed, particularly for links common to both rotation modes and those unique to CW rotation.

Encouraged by our success in recovering the biologically meaningful conformation of the FliN homodimer using only coevolutionary signals, we decided to test the viability of a biologically relevant FliM_*C*_–FliN_*C*_ heterodimer. The FliM_*C*_ monomer for *T. maritima* was modelled using the sequence corresponding to the FliMN_*C*_ domain and the previously cited crystallographic data to the FliN_*C*_ chain A as a template (PDB ID: 1O6A). DCA couplings from the FliMN_*C*_ family were also mapped onto the FliM_*C*_ sequence, and the resulting predicted coevolving interfacial residues between FliN_*C*_ and FliM_*C*_ were used to drive the complex formation of this heterodimer. The interface of the resulting complex (figure 3*a*; electronic supplementary material, figure S5 and Movie S2) is consistent with previous studies [67]. The geometry is preserved, and the interface we recover agrees accurately with the experimental evidence [67], with an interfacial structural alignment with RMSD of 1.31 Å when compared with a fusion protein comprising both portions of FliM_*C*_ and FliN_*C*_ from *S. enterica* (PDB ID: 4YXC).

The reported model describing the lower part of the C-ring as an array of closed-ring-like FliN_*C*_ homotetramers intercalated by FliM_*C*_ monomers [22] is supported by the interpretation of cross-linking data and observed mutational effects. In order to evaluate the agreement of our predicted heterodimer with these data, we compared the reported cross-linking experiments between FliM_*C*_ and FliN_*C*_ with the interfacial contacts of this complex. Figure 3*b* shows that the complex predicted for FliM_*C*_ and FliN_*C*_ presents a different interface from the one originally proposed based on experimental data [22]. In the predicted heterodimer, instead of a side-to-side interaction between FliN_*C*_ and FliM_*C*_ that would be compatible with the idea of FliN_*C*_ homotetramers intercalated by single FliM_*C*_ monomers, we observe a heterodimer conformation that shares the same interface of that observed in FliN_*C*_ homodimers. This possibility of a common dimerization interface for FliN_*C*_ and FliM_*C*_–FliN_*C*_ was recently evidenced by other studies [16]. Remarkably, even though the predicted binding mode differs from the one originally proposed based on experimental data, it satisfies the related observed cross-links between FliM_*C*_ and FliN_*C*_, with dimeric interacting pairs coinciding with the regions of most cross-linking occurring in both FM directional rotation modes (red points in figure 3*b*). Furthermore, this correspondence is more evident when we consider the agreement between cross-linking data observed only in the FM CW rotation mode, as denoted by the green point in figure 3*b*. Moreover, it has been reported that specific mutations in FliN_*C*_ are able to prevent its binding to FliM_*C*_ [22]. Electronic supplementary material, figure S6 shows that most of these residues (five out of six, depicted in green) are accurately located at the interface of the proposed complex, where they contribute to dimer stabilization. Altogether, these results support the idea of a coexistence of functional FliN_*C*_ homodimers and FliM_*C*_–FliN_*C*_ heterodimers, as proposed by McDowell *et al.* [16].

### 3.3. FliM oligomerization and interface switching

**Figure 4.**
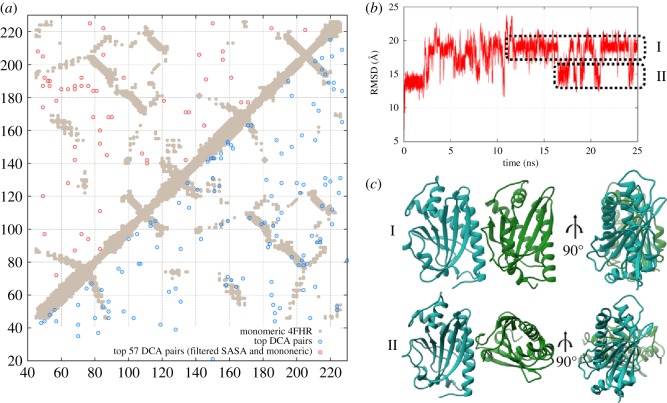
(*a*) Coevolutionary analysis of the FliM_*M*_ domain family. DCA contacts (blue circles) reproduced the monomeric contacts occurring in the FliM_*M*_ crystal structure (beige dots, PDB ID: 4FHR) [70]. Dimeric contacts (red circles) were selected from the combined monomeric/dimeric signals by excluding solvent inaccessible and monomeric contacts. (*b*) MD simulation using DCA contacts to predict FliM_*M*_ homo-dimeric complex formation. At the final stage of the simulation, an oscillation of dimeric complexes was observed in two distinct modes (I and II). (*c*) In dimeric model I for FliM_*M*_, monomers interact through side-to-side contacts to promote a parallel configuration. In model II, monomers interact in a twisted configuration.

FliM is one of the fundamental components of the flagellar basal body. Its homo-oligomerization in a 34-fold ring is reported to constitute the central core of the C-ring (figure 1), whereas the outer rings are formed mainly by FliN and FliG [68–71]. Several studies suggest FliM is directly involved in C-ring conformational changes related to the direction of flagellar rotation [53,68,69,71]. Furthermore, cryo-electron microscopy and cross-linking experiments point out a possible side-to-side symmetrical association among FliM subunits that could explain ring oligomerization, but molecular details about the interfaces for this multi-complex still remain unsolved [4,21,69,70]. In order to uncover the most plausible oligomerization interface for FliM involved in the C-ring formation, we analysed the family of the middle domain of FliM (FliM_*M*_) to extract evolutionary hints about dimerization to be able to infer complex formation using MD simulations with SBMs. As a first step, we identified a relatively abundant set of FliM homologues (see Material and methods section for details) with 3939 sequences corresponding to FliM_*M*_ (Pfam PF02154). The MSA of this dataset served as the basis for coevolutionary analysis with DCA. The identified coevolved residues at the interface were then mapped onto the *T. maritima* FliM sequence, and the predicted contacts were compared with those reported on a crystal structure from PDB 4FHR (figure 4*a*). We recovered clear signals from monomeric contacts, but we also observed clusters of reside–residue correlations outside of the monomeric conformation. Following our methodology for dimerization prediction, signals from non-solvent accessible residue pairs and interaction pairs related to folding were excluded. The remaining directly correlated residue pairs (figure 4*a*) were then used in a simulation to drive the dimerization of two monomeric FliM_*M*_ structures using chain A of the structure 4FHR (see Material and methods section).

**Figure 5.**
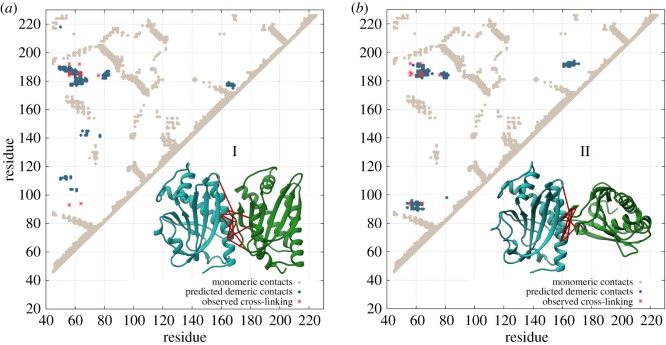
Dimeric contacts at the interface of the predicted models for FliM_*M*_ oligomerization. (*a*) Model I, in a parallel side-to-side configuration and (*b*) model II, in a twisted perpendicular configuration. Both models present an equivalent number of contacts and a common interface interaction region that highly correlates with experimental data from cross-linking experiments (red connections in three-dimensional models) [69].

We computed MD trajectories for two FliM_*M*_ monomers using highly coupled evolutionary pairs as interaction constraints in our computational model. Figure 4*b* shows the RMSD for the trajectory of the complex formation simulation. In the last stage of the simulation, two interaction modes appear to dominate the conformational space by switching of the dimeric interface in a dual interaction mode (figure 4*b*; electronic supplementary material, Movie S3). Clustering trajectories in both modes allowed us to identify two distinct binding interfaces, characterized by a parallel and a twisted orientation between each FliM_*M*_ monomer (figure 4*c*). A three-dimensional depiction of representative DCA pairs that promoted both twisted and parallel conformations is shown in electronic supplementary material, figure S7. Comparison of the dimeric contacts formed in each configuration showed that residues involved in complex formation have a common cluster region in the quadrant formed by residues 50–80 and 175–190 (figure 5). Furthermore, interfaces I and II both consist of about the same number of contacts, with further distinct secondary clusters of dimeric contacts. We have observed similar features of conformational plasticity in ligand-binding proteins using coevolutionary information, where functional modes are encoded by evolution [31]. This is the first time that we have observed similar features for protein complexes.

In order to provide further evidence for the validity of our predictions, we compared the resulting dimeric contacts with reported data for cross-linking experiments with FliM_*M*_ oligomers [69]. Figure 5 shows that the most populated region of predicted dimeric contacts in both complexes is located exactly at the interface of previously reported cross-linking pairs (see red markers) related to oligomerization [8,21,69]. Remarkably, although model I agrees very well with the parallel side-to-side FliM_*M*_ oligomerization suggested by EM density map fitting [70], model II with a twisted dimerization interface is the one that accurately satisfies all reported cross-linking evidence, with a secondary interface region exactly superposing two disulfide cross-linking Cys pairs (56/93 and 64/94) from a mutant FliM_*M*_ [69]. This higher correspondence between cross-linking and the predicted interface can also be observed as shorter pairwise distances in the three-dimensional representation of model II when compared to model I (figure 5).

It is notable that although the parallel binding mode is the one that best fits the EM map [4] (representing CW rotation state), the perpendicular mode predicted in this study is the one that accurately satisfies all cross-linking data. When we measure the distance between C*α* atoms in our proposed models for all the cross-linking pairs, we observe that the perpendicular model has on average smaller distances compared with the parallel model (electronic supplementary material, table S1) [69]. In addition, both predicted complex configurations were independently obtained based solely on coevolutionary signals. Together, these facts suggest that these parallel and twisted predicted interfaces can represent functional conformations of a FliM switching mechanism related to CW and CCW directional rotation of FM, respectively.

## 4. Discussion

FliN_*C*_ and FliM_*C*_ interact to form the membrane-distal portion of the C-ring in flagellar motors. However, the precise framework and interfaces involved in the association of these proteins is still a source of questions. While some studies suggest this region is composed of FliN_*C*_ homotetramers intercalated by FliM_*C*_ monomers [22], other results indicate a common functional dimerization interface between FliN_*C*_ and FliM_*C*_ [16]. Despite these findings, only chimeric constructions with fused FliM_*C*_ and FliN_*C*_ domains structure have been reported [67]. Furthermore, the molecular details of FliM oligomerization to build the central portion of C-ring and its conformation involved in FM switching are still elusive. Amino acid coevolution has been in the spotlight as a breakthrough methodology to identify molecular interactions when experimental measurements are inadequate. Methods using coevolution have finally reached a mature state that allows us to make predictions rather than focusing on validations. Moreover, we have developed an efficient methodology to identify interfaces involved in complex formation [45]. Here, we have shown a biologically relevant application of this approach to predict precisely the interface involved in the formation of FliN homodimers (figure 2). Furthermore, we show that, using our methodology, coevolutionary signals between FliM_*C*_ and FliN_*C*_ proteins are able to generate a stable heterodimer (figure 3) that shares features found in FliN_*C*_ homodimers. The possibility of reproducing this conformation entirely from coevolution suggests that this heterodimer is a functional complex and supports the idea that FliN_*C*_ and FliM_*C*_ share the same interaction interface. This interaction pattern could lead to the spiral-like framework formed by the repetitive association of FliM_*C*_–FliN_*C*_ heterotetramers in a proportion of 1 : 3, as proposed by McDowell *et al.* [16]. The predicted model for FliM_*C*_–FliN_*C*_ heterodimer is also in conformity with FliN_*C*_ mutations and FliM_*C*_–FliN_*C*_ disulfide cross-linking data that support the FliN_*C*_ doughnut-shaped homotetramer model (figure 3*a*; electronic supplementary material figure S6) [22].

FliM is located in the central part of the C-ring and plays a key role in mediating signal propagation for switching of FM directional rotation through conformational changes. Currently, there are only models for FliM_*M*_ oligomerization, generated from EM density fitting [70,71] and sparse cross-linking data [69]. This model describes FliM_*M*_ monomers arranged in a parallel side-to-side orientation, forming a symmetrical ring-like framework, although the details of their interface for oligomerization remains unclear. Predictions of FliM_*M*_ complexes using coevolutionary information allowed us to identify two stable binding interfaces that are able to switch between themselves (figure 4*b*,*c*). While one of these homodimer models (figure 5*a*) agrees very well with the arrangement of parallel side-to-side FliM_*M*_ monomers and satisfies a fraction of the reported FliM disulfide cross-links [69], a second, twisted model, in which both monomers interact in a perpendicular orientation presents interface contacts that reproduce precisely those observed in disulfide cross-links experiments (figure 5*b*). Moreover, this predicted twisted interface involves contacts over a unique region encompassing residues 160–170 and 190–195 of FliM (figure 5*b*). Therefore, alternative cross-linking experiments involving this region in wild-type FliM (e.g. zero-length pairing of Ser167 and Glu193) could provide additional evidence to corroborate this alternative conformational state, as well as the correspondence between the predicted interface and the cross-linking data.

The observation of dimeric contacts that match exactly to experimental cross-linking that are not seen in the suggested parallel FliM_*M*_ dimerization model [69,70] (cross-links at residues 56/93 and 64/94) suggests that the twisted FliM_*M*_ complex identified (figure 5*b*) could depict a novel conformational state of the basal body when the FM is in CCW flagellar rotation. We believe that the conformation shown in model II might be connected with a transient conformational state and could represent a strained ring conformation. FliM has an *α*/*β*/*α* domain with considerable flexibility, as we showed in an early study [53]. Contacts formed by adjacent FliG, FliM (C-terminal) and FliN proteins could lock-in into a strained ring state consistent with the dynamic loss/gain of FliM subunits. We also envision rings where not all the dimeric interfaces are identical; it is possible to find a mixture of interfaces *in vivo* where model I and model II coexist. Possible more-intricate interactions should also be considered in this system, such as the intercalation of identical dimers by switching of activation factors and the simultaneous presence of both models I and II. This information could provide further insights into the overall molecular mechanism involved in the FM switching system. Together, these studies shed light on unresolved questions about the formation of complex structures in flagellar systems. They suggest novel dynamic properties of FliM dimerization that can be tested experimentally. We hope that our results can serve as a basis for further research aimed at unraveling more molecular details about the switching mechanisms of bacterial flagellar motors.

## Supplementary Material

Supplementary Figure S1

## Supplementary Material

Supplementary Figure S2

## Supplementary Material

Supplementary Figure S3

## Supplementary Material

Supplementary Figure S4

## Supplementary Material

Supplementary Figure S5

## Supplementary Material

Supplementary Figure S6

## Supplementary Material

Supplementary Figure S7

## Supplementary Material

Supplementary Table S1

## Supplementary Material

Supplementary Table S2

## Data accessibility

All research materials supporting the results of studies reported in this manuscript are available in the electronic supplementary material, consisting of figures, tables and simulation trajectory movies. All raw metadata used to derive the studies are deposited in Dryad Digital Repository: (http://dx.doi.org/10.5061/dryad.0mv6t) [72].
