# Supplementary figures and images for: Characterization of C-ring component assembly in flagellar motors from amino acid coevolution
Source: R Soc Open Sci. 2018 May 9;5(5):171854. doi: 10.1098/rsos.171854 (PMC5990795; doi:10.1098/rsos.171854)

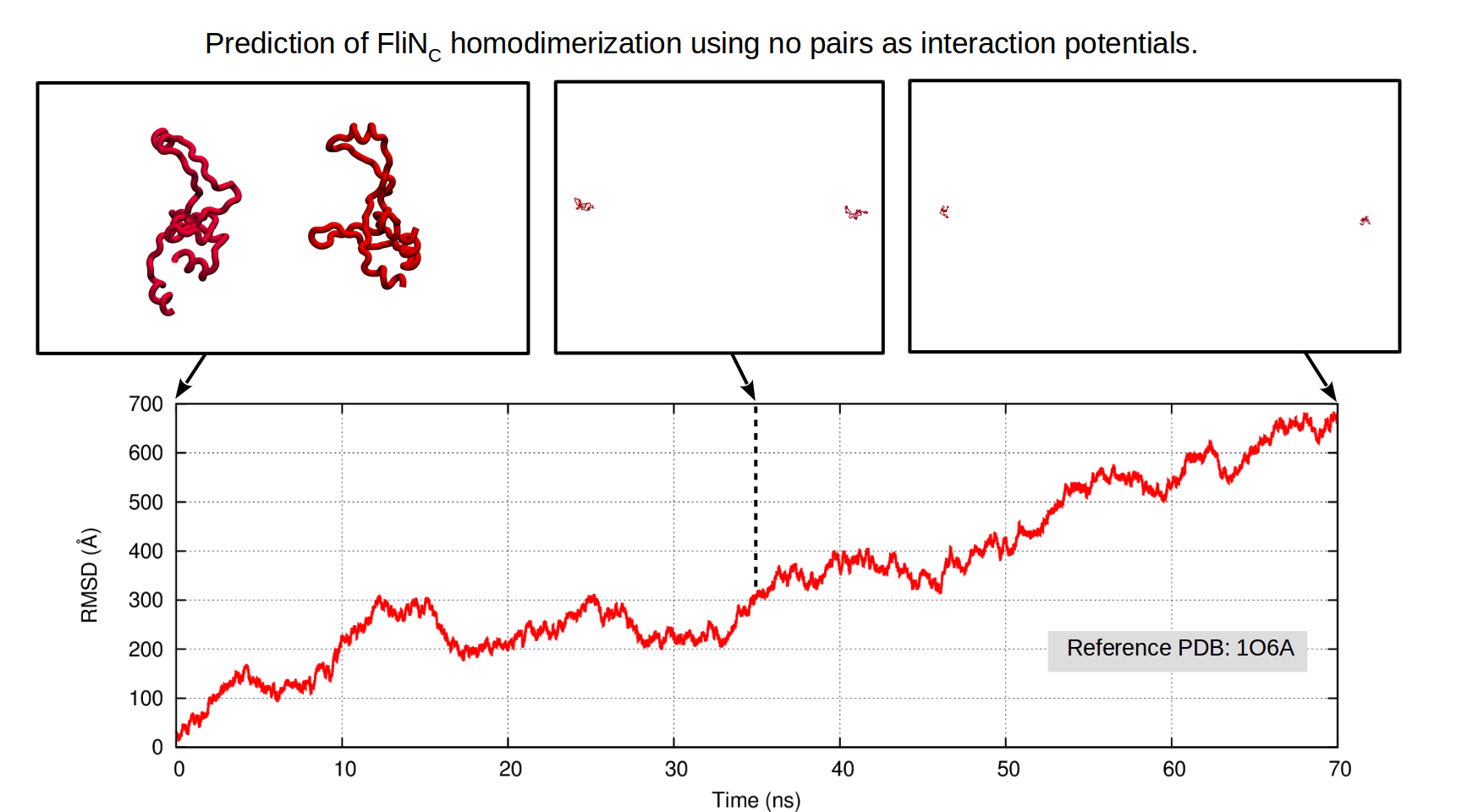

Supplement: Supplementary Figure S1 [file rsos171854supp1.tiff]

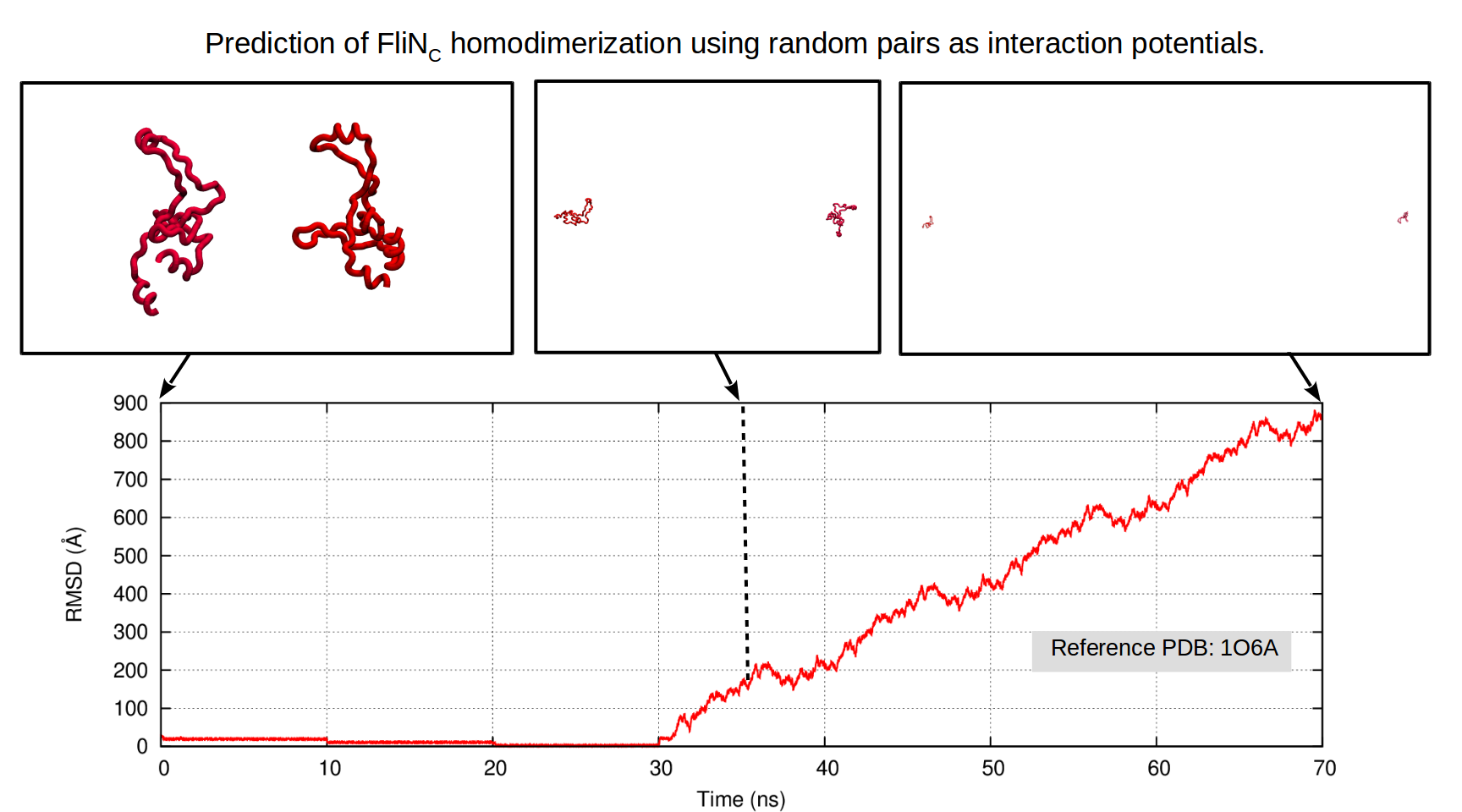

Supplement: Supplementary Figure S2 [file rsos171854supp2.tiff]

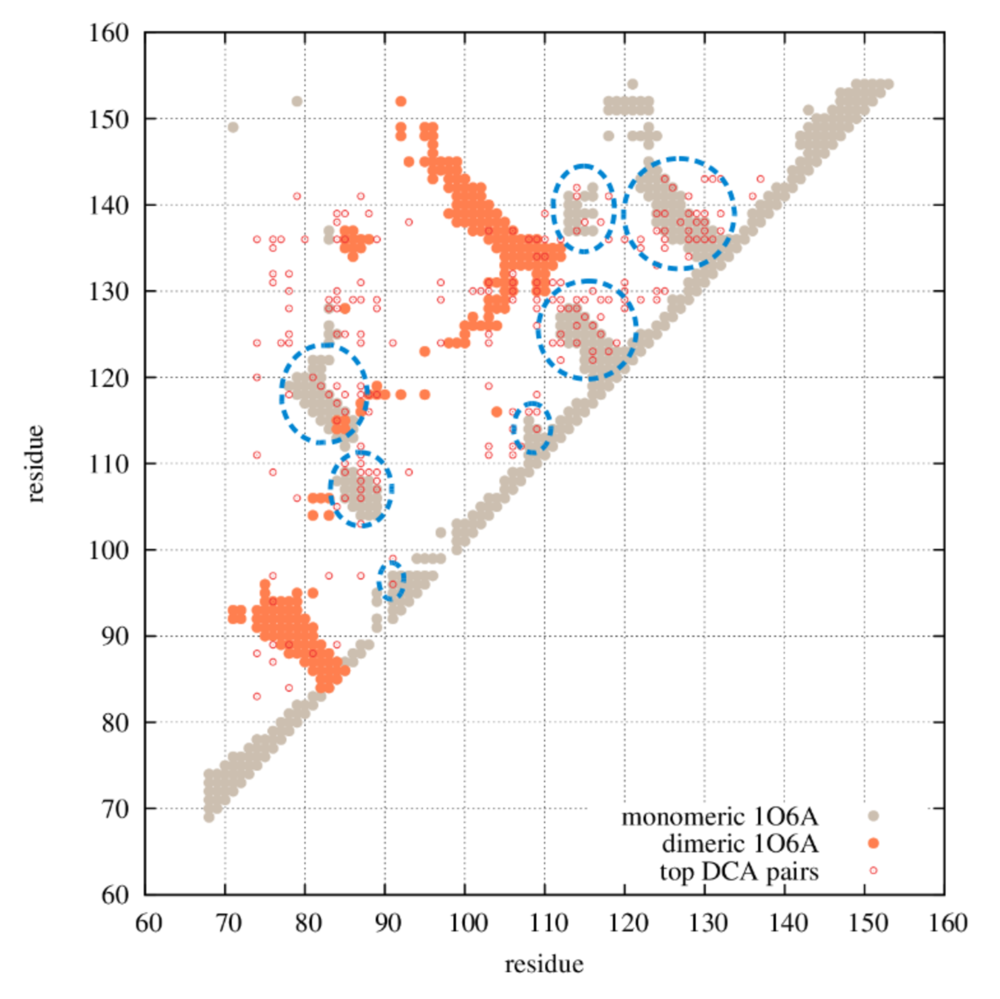

Supplement: Supplementary Figure S3 [file rsos171854supp3.tiff]

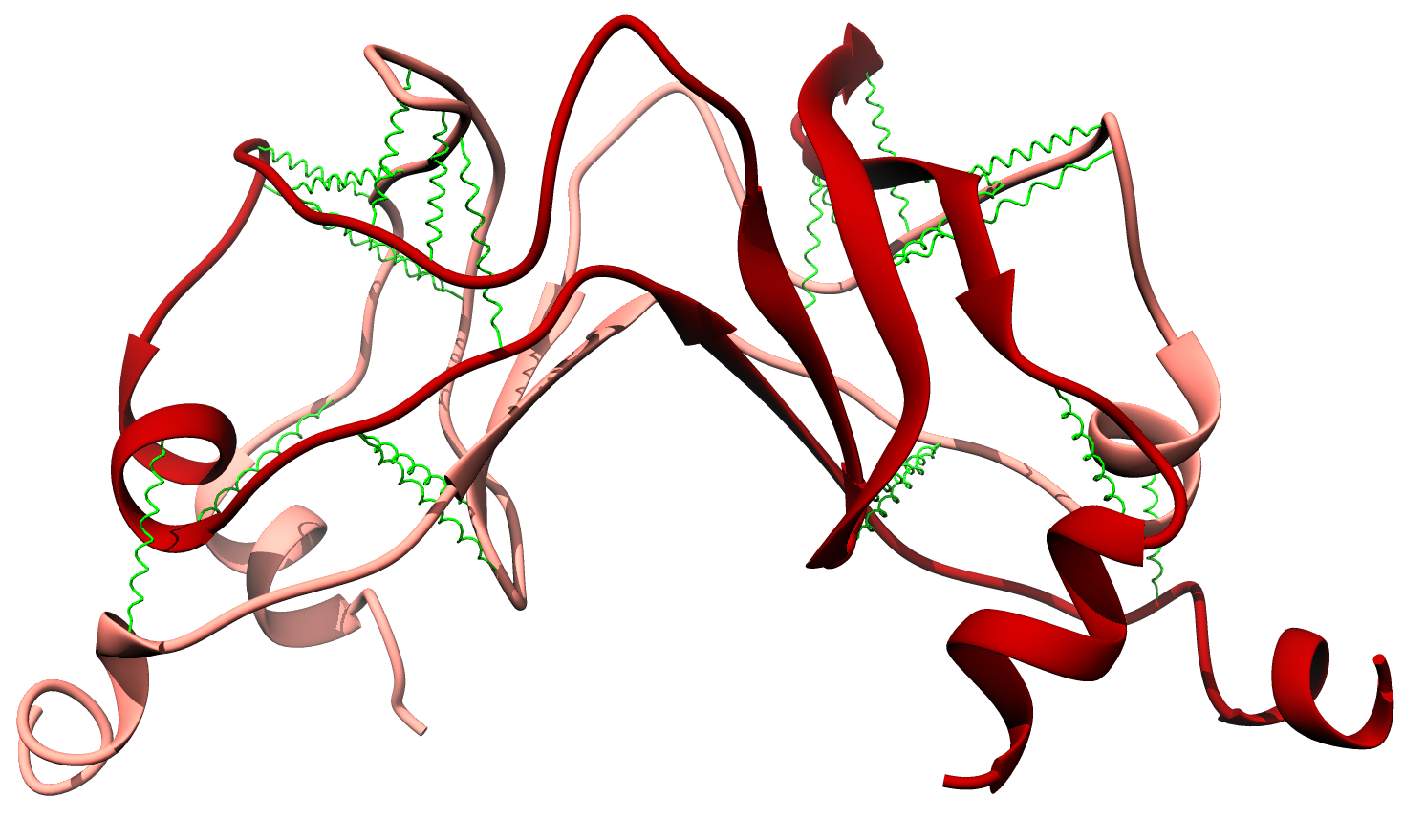

Supplement: Supplementary Figure S4 [file rsos171854supp4.tiff]

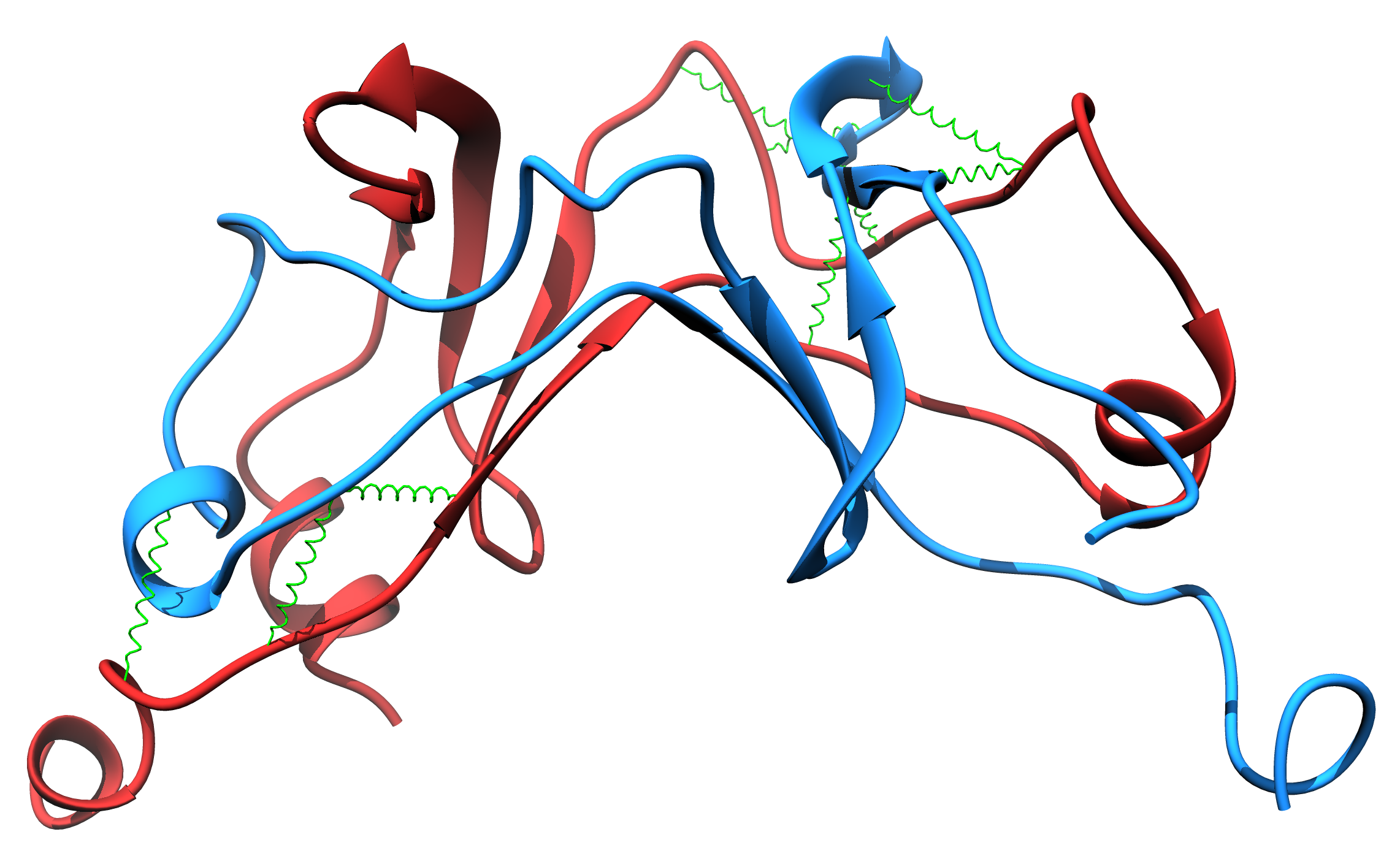

Supplement: Supplementary Figure S5 [file rsos171854supp5.tiff]

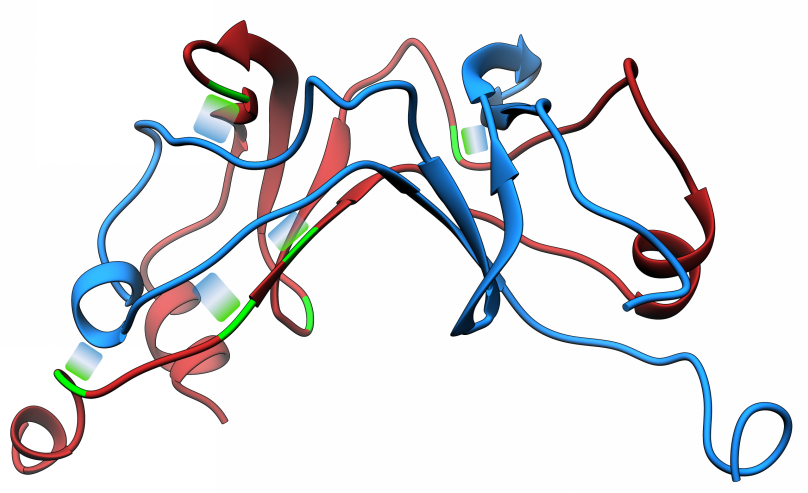

Supplement: Supplementary Figure S6 [file rsos171854supp6.tiff]

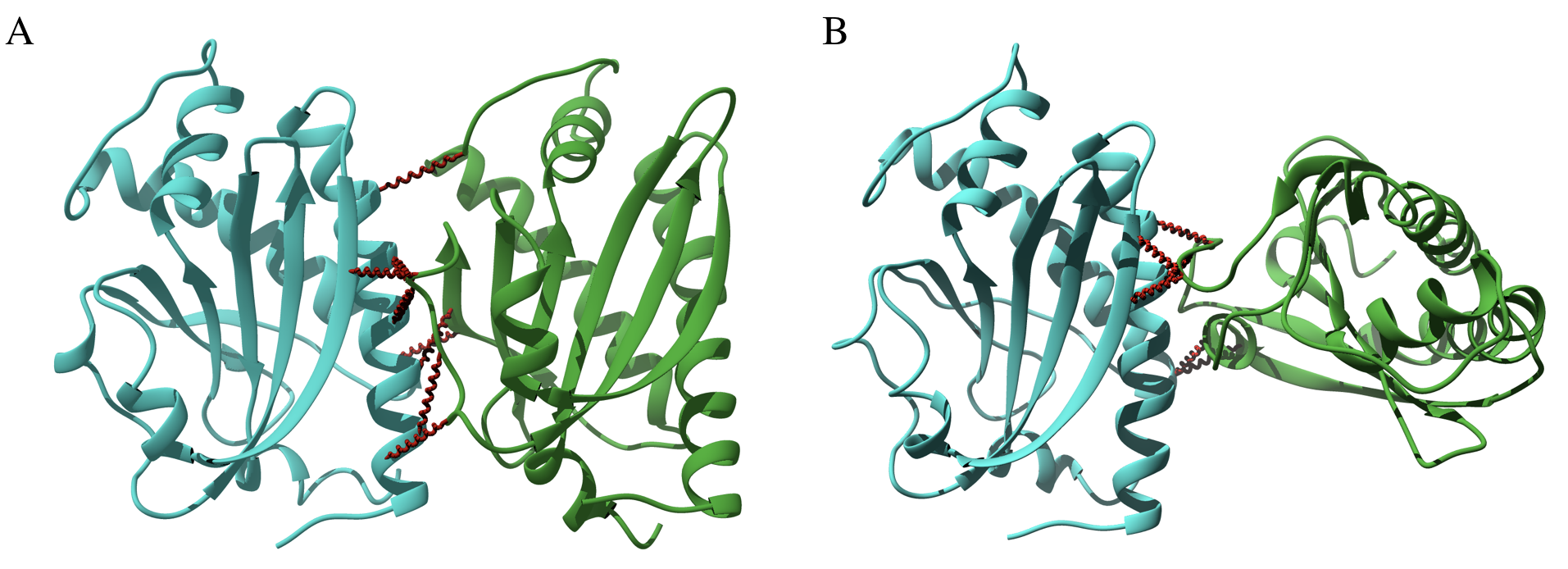

Supplement: Supplementary Figure S7 [file rsos171854supp7.tiff]
