# Supplementary material for: Characterization of C-ring component assembly in flagellar motors from amino acid coevolution
Source: R Soc Open Sci. 2018 May 9;5(5):171854. doi: 10.1098/rsos.171854 (PMC5990795; doi:10.1098/rsos.171854)
Supplement: Supplementary Table S1 [file rsos171854supp8.pdf]

Table S1: Comparison of predicted $FliM_M$ complexes with reported cross-link signals.

| pair | distance $C\alpha$-$C\alpha$ (Å) | |
|---|---|---|
| | Model I | Model II |
| 57/94 | 16.4 | 5.9 |
| 57/185 | 8.0 | 14.7 |
| 57/187 | 5.9 | 14.2 |
| 64/94 | 17.0 | 5.9 |
| 64/185 | 6.7 | 5.8 |
| 64/187 | 12.3 | 5.7 |
| 77/185 | 15.7 | 8.3 |

Comparison of predicted $FliM_M$ complexes with reported cross-link signals.
