# Supplementary material for: Characterization of C-ring component assembly in flagellar motors from amino acid coevolution
Source: R Soc Open Sci. 2018 May 9;5(5):171854. doi: 10.1098/rsos.171854 (PMC5990795; doi:10.1098/rsos.171854)
Supplement: Supplementary Table S2 [file rsos171854supp9.pdf]

Table S2: Parameters for complex predictions using SBM molecular simulations.

monomer 1	monomer 2	DCA restraints	RMSD (Å)
$FliN_C$	$FliN_C$	200	0.81
$FliN_C$	$FliN_M$	200	1.44*
$FliM_M$	$FliM_M$	57	-

* Interfacial RMSD (iRMSD) from structural alignment considering a chimeric X-ray model of fusioned $FliN_C$ and $FliM_C$ fragments from Salmonella enterica (PDB ID: 4YXC).

Parameters for complex predictions using SBM molecular simulations.
